# Evaluating the physical and psychosocial impact of serious physical combat injuries in UK armed forces personnel-the ADVANCE cohort study

**DOI:** 10.1007/s10654-025-01300-2

**Published:** 2025-09-24

**Authors:** Daniel Dyball, Susie Schofield, Howard Burdett, Christopher J. Boos, Anthony M. J. Bull, Paul Cullinan, Nicola T. Fear, Alexander N. Bennett, ADVANCE Study, Helen Blackman, Grace Blissitt, Melanie Chesnokov, Emma Coady, Sarah Evans, Guy Fraser, Nicola Goodman, Alison Hever, Meliha Kaya-Barge, Jocelyn Keshet-Price, Maija Maskuniitty, Eleanor Miller, Steven Parkes, Bharti Patel, Samatha Paul, David Pernet, Vlad Pop, Helen Prentice, Urszula Pucilowska, Stefan Sprinkmoller, Jodie Stevenson, Lalji Varsani, Anna Verey, Molly Waldron, Owen Walker, Danny Weston, Tasarla White, Seamus Wilson

**Affiliations:** 1 King’s Centre for Military Health Research, King’s College London, London, SE5 9RJ UK; 2 National Heart and Lung Institute, Faculty of Medicine, Imperial College London, London, SW3 6LR UK; 3 Faculty of Health & Social Sciences, Bournemouth University, Bournemouth, BH1 3LT UK; 4 Centre for Injury Studies, Department of Bioengineering, Imperial College London, London, SW7 2AZ UK; 5 London, UK; 6 Academic Department of Military Mental Health, King’s College London, London, SE5 9RJ UK; 7 Academic Department of Military Rehabilitation, Defence Medical Rehabilitation Centre, Stanford Hall Estate, near Loughborough, Nottinghamshire, LE12 5BL UK

**Keywords:** ADVANCE cohort, Wounds and injuries, Mental health, Heart disease risk factors, Disease, Epidemiologic studies, Military

## Abstract

The ADVANCE cohort study is a prospective cohort study investigating the impact of sustaining a serious physical combat injury whilst on deployment to Afghanistan on long-term health outcomes. The cohort will provide essential data on medical/psychosocial risk factors and outcomes associated with combat injury and establish injury-specific mechanisms of disease. Participants include UK Armed Forces personnel who sustained serious physical combat injuries and a frequency-matched comparison group who sustained no such injuries (uninjured group). The cohort consists of 1145 participants, with a baseline response rate of 59.6% for the injured group (*n* = 579) and 56.3% for the uninjured group (*n* = 566). The first follow up of this cohort retained 92% of the sample (*n* = 1053/1145). This cohort profile describes the baseline and first follow up demographics for ADVANCE, as well as details on published research projects spanning military epidemiology, physical health, including cardiovascular, respiratory, musculoskeletal and neurological, mental health and health-related behaviours. These projects comprise identification of early risk factors for disease and observed differences in health-characteristics between groups. Since baseline assessment of the cohort, ADVANCE has expanded investigations across physical and mental health domains, utilising advice from participant engagement and an international scientific advisory group. Researchers working on the ADVANCE cohort continue to engage with policy makers, clinicians and participants to ensure a wide-ranging impact from work conducted.

*Registration*: The ADVANCE Study is registered at ISRCTN ID: ISRCTN57285353.

**Supplementary Information:**

The online version contains supplementary material available at 10.1007/s10654-025-01300-2.

## Introduction

Due to advances in medical and defence technology over the period of the Iraq and Afghanistan conflicts (2001–2014), more UK military personnel who sustained serious physical injuries during their deployment to Afghanistan survived compared to any other recent conflict [1, 2]. The long-term impact on health and well-being for those who survived such injuries, including those with associated limb-loss, are currently unknown.

The ArmeD serVices trAuma rehabilitatioN outcomE (ADVANCE) study is a prospective cohort study designed to examine the long-term health impact of sustaining a serious physical combat injury during deployment to Afghanistan (2003–2014; Operation HERRICK) [3]. The primary hypothesis of ADVANCE is that sustaining serious physical combat injuries will be associated with increased incidence of adverse cardiovascular and musculoskeletal outcomes compared to demographically similar military personnel who did not sustain such injuries. Secondary hypotheses are linked to increased risk of other adverse medical, psychosocial, and vocational outcomes.

## Cohort description

Potential participants who sustained serious physical combat injuries were identified based on Ministry of Defence records provided by the department of Defence Statistics [3]. All injured personnel were aeromedically evacuated to a UK hospital and included personnel that sustained injuries with associated limb loss, those that were Very Seriously Injured (VSI) or Seriously Injured (SI) and a random sample of personnel with unclassified or ‘other’ injuries that required aeromedical evacuation [3]. A comparison group of individuals who did not sustain serious physical combat injuries were also identified by Defence Statistics (herein called the ‘uninjured’ group). These were frequency-matched to the injured group based on age, specific deployment period (i.e. HERRICK 1-HERRICK 20), rank whilst on deployment, role on deployment, service branch and regiment.

Power calculations were based on a primary composite cardiovascular disease event rate of > 10% in the uninjured group and 17% in the injured group (13.5% overall) at endpoint of the study (20 years from baseline assessment). A sample size of at least 413 both the injured and uninjured group would have > 80% power to detect a hazard ratio of > = 1.7 [3]. A recruitment pool of 1400 participants with eligible injuries and 2100 demographically similar personnel without eligible injuries were identified by Defence Statistics, of which a random sample of each was approached to take part.

### Baseline recruitment

**Fig. 1:**
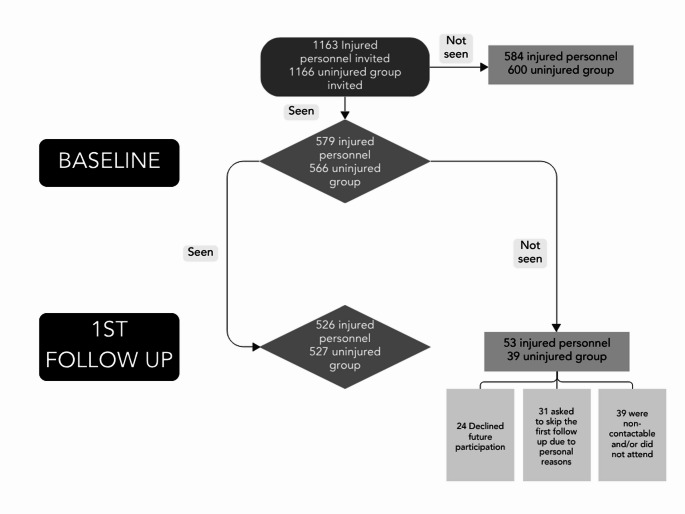
Baseline and follow up recruitment

The study began in August 2015 and baseline recruitment of participants was completed in August 2020 [4]. Participants were approached using a combination of post, email, telephone, social media, engagement with military bases and military charities, and tracing via electoral roll data. Informed consent was collected from participants at the baseline assessment for use of data gathered for research purposes, future contact to take part in additional assessments and NHS medical records data linkage. Overall, 579 participants were recruited to the injured group and 566 to the uninjured group (Fig. 1). A response rate, adjusted for deaths and potential participants without means of contact, of 59.6% (579/971) was achieved for the injured group and 56.3% (566/1005) for the uninjured group. Of the injured group, 55.1% of eligible VSI or SI took part, and 62.6% of those who sustained an eligible injury with associated limb loss (amputation injury subgroup) took part.

### First follow up recruitment

Participants who took part in baseline were invited to take part in additional follow ups, having previously given their consent. The first follow up of participants started in August 2018 and finished in August 2023. Details regarding the baseline demographics and attrition rate can be found in Table 1; Fig. 1. Of those that took part in the baseline assessment, ADVANCE achieved a retention rate of 90.8% (526/579) for the overall injured group and 93.3% (527/565) for the uninjured group. Due to difficulty attending the follow up as a result of e.g. work, health, or being out of the country, some individuals were unable to attend, but did not wish to decline participation long-term. Active declines represent only a very small number of individuals in each group: 16/579 (2.8%) of participants in the injured group and 8/565 (1.4%) of participants in the uninjured group. At point of injury/deployment of interest (sampling), participants who attended the first follow up assessment had a median age of 25, were predominantly junior non-commissioned officers/other ranks (64.4%) and predominantly served in the Army (82.8%). At point of follow up assessment, participants had a median age of 37.

Demographics of non-responders compared to responders can be found in supplementary materials 1. Non-responders, including those who did not attend their first follow up but did not actively decline as well as those that actively declined, were a median age of 24 and were predominantly junior non-commissioned officers/other rank (82.6% of non-responders) and served in the Army (81.5%) at point of injury/deployment of interest (sampling). A logistic regression analysis of non-response to the first follow up of ADVANCE indicated that, compared to the uninjured group, there was no difference in the odds of not responding to the first follow up for the amputation injury subgroup (Odds Ratio (OR) 1.76, 95% Confidence Interval (CI) 0.96, 3.05) or injuries without associated limb loss (non-amputation injury subgroup) (OR 1.09, 95%CI 0.62, 1.67) (supplementary materials 2). As age increased, the odds of not-responding decreased (OR 0.93, 95%CI 0.88, 0.98), indicating that younger individuals were significantly less likely to attend the first follow up.

### Future follow up

The second follow up of participants started in 2023 and is planned to finish in 2026. Everyone who took part in baseline, excepting those who actively declined further participation, will be approached. Additional follow ups are planned up to 20 years from baseline assessment.

### Participant engagement

Participants are invited to follow ADVANCE on social media, which is utilised alongside quarterly newsletters to keep followers up to date with study activities (such as the beginning of new follow up phases) and new publications. All papers as well as one-page lay summaries are made available on our website (www.advancestudydmrc.org.uk/publications).

In 2021, ADVANCE hosted their first participant panel. The panel consists of a range of individuals with/without having experienced serious physical combat injuries. The panel gives personal points of view on upcoming papers, achieving societal impact from the study results, the direction of ADVANCE and future avenues of research.

### Independent scientific advisory group

In 2023, an independent international scientific advisory group was established for the ADVANCE study. The group acts as a critical friend to the study and is chaired by a highly experienced clinical epidemiologist alongside 12 members, with expertise spanning epidemiology, cardiovascular disease, mental health, neurology/neuroscience, social research, military health, expert patients and lay members.

### Data analysis: weights

Sampling weights, to take into account the under-sampling of the less seriously injured group, and response weights, based on the inverse probability of responding, were calculated for the baseline assessment. Using a logistic regression model including age, rank and service at point of sampling we calculated response weights through the inverse of the predicted probabilities of responding. Response weights were multiplied by sampling weights to create an overall baseline weight. For the first follow up, an attrition weight was calculated using data collected at baseline. LASSO was used to reduce the model. This logistic regression model included smoking status, age, combat role, rank and relationship status. A longitudinal weight for the first follow-up was calculated by multiplying the attrition weight at the first follow up by the overall baseline weight.

**Table 1:** Baseline demographics

	Serious physical combat injury group		Uninjured group	
	Characteristics at Baseline (*n* = 579)	Characteristics at First follow up (*n* = 526)	Characteristics at Baseline (*n* = 566)	Characteristics at First follow up (*n* = 527)
Time from sampling to assessment* in years (median (Interquartile range)	8.2 (6.9, 9.7)	11.6 (10.2, 13.0)	7.6 (6.6, 9.1)	11.2 (10.0, 12.5)
Age at baseline assessment in years (median (Interquartile range))	33 (30, 37)	33 (30, 37)	34 (30, 37)	34 (30, 37)
Age at follow up assessment in years (median (Interquartile range))	-	37 (34, 41)	-	38 (34, 41)
Rank (n (%))				
Other rank/junior non-commissioned officers	414 (71.5%)	368 (70.0%)	340 (60.0%)	310 (58.8%)
Senior non-commissioned officers	106 (18.3%)	100 (19.0%)	147 (26.0%)	142 (26.9%)
Officer rank	59 (10.2%)	58 (11.0%)	79 (14.0%)	75 (14.2%)
Service (n (%))				
Naval Services (including Royal Marines)	77 (13.3%)	66 (12.6%)	84 (14.8%)	80 (15.2%)
Army	484 (83.6%)	443 (84.2%)	463 (81.8%)	429 (81.4%)
Royal Airforce	18 (3.1%)	17 (3.2%)	19 (3.4%)	18 (3.4%)
Type of injury (n (%))				
Amputation injury	161 (27.8%)	141 (25.0%)	-	-
Non-amputation injury	418 (72.2%)	385 (75.0%)	-	-
New Injury Severity Score (median (Interquartile range))	12 (5, 22)	12 (5, 22)	-	-

*Calculated from the difference between age at index injury for injured personnel or age at sampled deployment + 0.5 (to reflect average age during sampled year) for the uninjured group and age at baseline/follow up assessment. Weighted percentages are presented alongside unweighted cell counts

.

### Measures

Information is collected from a comprehensive suite of health assessments, including clinical interview, self-report questionnaire and medical investigations. Military deployment-related data at point of sampling, including data from the Joint Theatre Trauma Registry (JTTR), was also provided by Defence Statistics for all participants who consented to take part. Table 2 describes the information collected, the method of collection and the differences in information collected between baseline and future assessments.

**Table 2:** Types of data collected

	Baseline Assessment					First Follow up					Second Follow up				
	CI	Q	MOD	ME	A	CI	Q	MOD	ME	A	CI	Q	MOD	ME	A
Sociodemographic															
Basic demographics (e.g. age, ethnicity)	✓	✓	✓			✓	✓				✓	✓			
Family (e.g. carer status, relationship status family dynamics)		✓					✓					✓			
Financial wellbeing												✓			
Occupation/Education		✓					✓					✓			
Health-related behaviours															
Exercise		✓					✓					✓			✓
Legal/Illegal substance use		✓					✓					✓			
Sleep		✓					✓					✓			✓
Military history															
Deployment history			✓				✓					✓			
Military demographics (e.g. veteran status, rank)	✓	✓	✓			✓	✓				✓	✓			
Physical health															
Biometrics	✓					✓					✓				
Cardiovascular	✓			✓		✓			✓		✓		✓		
Combat injury history	✓	✓	✓			✓	✓	✓			✓	✓	✓		
Hearing	✓			✓		✓			✓		✓			✓	
Haematology				✓					✓					✓	
Inflammation				✓					✓					✓	
Medications	✓	✓				✓	✓				✓	✓			
Musculoskeletal	✓	✓	✓	✓		✓	✓	✓	✓		✓	✓	✓	✓	
Past/family medical history	✓					✓					✓				
Physical functioning	✓	✓				✓	✓				✓	✓			
Physical health help-seeking (e.g. surgery, pain interventions)		✓					✓					✓			
Renal and liver functioning				✓					✓					✓	
Respiratory				✓					✓			✓		✓	
Surgery history	✓					✓					✓				
Traumatic Brain Injury						✓			✓		✓			✓	
Psychosocial															
Adverse childhood experiences		✓					✓								
Cognitive health						✓					✓				
Health-related quality of life		✓					✓					✓			
Mental health		✓					✓					✓			
Mental health help-seeking												✓			
Sexual functioning		✓					✓					✓			
Social support		✓					✓					✓			

*CI* clinical interview; *Q* Questionnaire; *MOD* ministry of defence records; *ME* medical examination; *A* Accelerometer

The study has also expanded its range of medical assessments over time. Magnetic Resonance Imaging (MRI), electrocardiogram, use of wearable actigraphy devices and measurement of venous blood proteomics are tools now utilised in follow up assessments.

#### Demographics/military history

Sociodemographic data and military data regarding deployment/combat injury was obtained from Defence Statistics, from medical records and Ministry of Defence records including the JTTR and Defence Medical Information Capability Programme; supplemented by data gathered from clinical interview and self-report questionnaire.

The study has expanded to investigate operational deployments after the UK withdrawal from Afghanistan (2014) and factors associated with transition from the military to civilian life, including education, employment, financial well-being, family structure and carers/caring responsibilities.

#### Health-related behaviours

Information on health-related behaviours has been collected throughout the study, including alcohol use, tobacco use, illegal drug use, prescription drug use, physical activity, sleep, and sexual health has primarily been collected via clinical interview and self-report questionnaire.

Since the baseline assessments, the study has expanded to investigate engagement in competitive sport/adventurous activities (e.g. INVICTUS games) and diet through self-report questionnaire. Wrist-based actigraphy data (GeneActiv, Activinsights), worn for 10 consecutive days after the ADVANCE assessment, is also collected as an indicator of physical activity levels and sleep health.

### Physical health

#### Cardiovascular/bloods

At baseline, information on cardiovascular health were collected via self-report questionnaire, clinical interview and medical examinations. Self-report/clinical interview data was primarily used to gather data on family history of cardiovascular disease, prescription medication history, past medical history and health-related behaviours (e.g. tobacco use). Medical examinations included venous blood sampling, Dual Energy X-ray Absorptiometry (DEXA), and carotid-femoral pulse wave velocity and brachial artery pulse waveform analysis (Vicorder^®^ ;Skidmore Medical, UK).

Since the baseline assessments, the study has expanded to investigate Heart Rate Variability (HRV) using a single lead electrocardiogram-based measurement (Mega Motion Faros 180 recorder®; Mega, Finland) and Q-RISK3 scoring.

### Musculoskeletal/physical functioning

At baseline, musculoskeletal outcomes were collected via self-report questionnaire, clinical interview and medical examinations. Self-report questionnaires were primarily used to assess disability and pain outcomes, as well as past medical history, surgery history, prescription medication, and health-related behaviours (e.g. exercise). Medical assessments included X-ray and DEXA scan to assess osteoarthritis, rheumatoid arthritis and bone mineral density.

Since the baseline assessments, the study has expanded to investigate multifaceted dimensions of pain, ranging from types of pain (e.g. neuropathic, chronic) to pain catastrophising. A subgroup of both those with amputation injuries and the uninjured group have been selected to conduct full gait analysis, lower limb MRIs and ultrasound.

### Neurological

At baseline, limited data was available on neurological health. Since the baseline assessments, the study has expanded to investigate lifetime history of Traumatic Brain Injury (TBI) and cognitive health [5]. The ADVANCE-TBI study began in May 2022, and utilises advanced MRI imaging (Phillips 3T Ingenia Elition® Magnetic Resonance Imaging (MRI) scanner), resting state functional-MRI, fluid biomarkers and neuropsychological assessments to assess brain structure and function. Clinical interviews and medical assessments are used to assess the history of head injury, post-concussion symptoms and a battery of neuropsychological assessments including tests of premorbid functioning, memory, processing speed, executive functioning, memory and reaction speeds [5].

### Respiratory

At baseline, prebronchodilator lung function measurements including forced expiratory volume in one second (FEV1) and forced vital capacity (FVC) were measured using the NDD EasyOne Connect spirometer® (NDD Medical, Switzerland).

Since the baseline assessments, the study has expanded to include a breathlessness questionnaire using the Medical Research Council (MRC) dyspnoea scale. A lung diffusion test (DLCO), to investigate gas transfer via diffusing capacity of lungs for carbon monoxide using the NDD EasyOne Pro pulmonary functioning device (NDD medical, Switzerland), was also introduced in the second follow up.

#### Psychosocial

At baseline, psychosocial outcomes were investigated via the use of self-report questionnaires and clinical interview. Self-report questionnaires were primarily used to assess mental health outcomes, including generalised anxiety, depression PTSD, and positive psychological functioning (e.g. Post-Traumatic Growth (PTG)). Additional psychosocial outcomes included relationship status, social support, adverse childhood events and sexual functioning.

Since the baseline assessments, the study has expanded to investigate links between combat injury, self-identity and appearance distress originally investigated as part of the UNITS study [6], as well as the use of therapeutic/charitable services originally investigated as part of the UK Adult Psychiatric Morbidity Survey [7].

#### What will be measured in the future?

Advances in medical technology may highlight new or emerging areas of interest. New investigations deemed appropriate and reasonable will be added at subsequent follow up assessments, including data linkages with NHS records. Data linkage with NHS records ensures that the study is sufficiently powered to address hypotheses regarding primary composite cardiovascular endpoints.

### Findings to date

All publications can be found on our website https://www.advancestudydmrc.org.uk/publications/.

### Physical health

ADVANCE has published research identifying that servicemen who have experienced serious physical combat injuries have a worse cardiovascular risk profile than that of demographically similar personnel who sustained no such injuries [8]. Features of their increased risk profile include greater relative abdominal obesity and visceral fat, systemic inflammatory response, arterial stiffness, lower HRV and dyslipidaemia [9–11]. Those injured have also been shown to have a higher prevalence of metabolic syndrome, estimated insulin resistance and indirect measures of myocardial blood flow reserve [8]. These are all subclinical cardiovascular risk markers which were relatively worse among the more severely injured and those injured with associated limb loss. Whether this risk will translate into worsening cardiovascular outcomes is the subject of ongoing follow up.

ADVANCE has identified that injuries with associated limb loss are accompanied by reduced bone mineral density [12], increased upper-limb disability [13], and a four-fold increase in odds of developing knee osteoarthritis compared to those who experienced no such injuries [14]. Three years later, Osteoarthritis rates across the cohort increased, with those with lower-limb loss at significantly higher risk, suggesting a shift from a post-traumatic to mechanoinflammatory mechanism [15]. Whilst different pathological processes may influence specific radiological features [16], no differences were seen in molecular patterns between those with post-traumatic and idiopathic osteoarthritis, suggesting a common mechanism, with relationships between adipokines and pain being present regardless of traumatic injury exposure, offering potential for phenotyping [17].

### Psychosocial

ADVANCE has published research identifying that sustaining a serious physical combat injury compared to sustaining no such injuries was associated with reporting probable anxiety (20.8% vs. 13.5%), depression (23.6% vs. 16.8%), PTSD (16.9% vs. 10.5%), mental health multimorbidity (15.3% vs. 9.8%) [4] suicidal ideation (15.3% vs. 11.9%) [18] and reporting the use of illegal drugs in the past year (16.3% vs. 5.4%) [19]. The authors found that individuals who experienced injuries without associated limb loss had greater odds of reporting mental illness [4] and greater risk of suicidal ideation [18] compared to the uninjured group. Individuals who experienced injuries with associated limb loss however, reported similar outcomes to the uninjured group. Veterans in both the injured and uninjured group reported greater rates of suicidal ideation and illegal drug use, with veterans in the uninjured group reporting the highest rates of both suicidal ideation [18] and illegal drug use [19].

PTG is the experience of beneficial psychological change following exposure to a trauma. ADVANCE has identified that personnel who experienced injuries with associated limb loss were more likely to report a large degree of PTG compared to the uninjured group, whereas those who experienced injuries without limb loss were not [20].

### Bridging the gap-physical and psychosocial

Higher frequency of moderate-severe pain was observed in individuals who experienced injuries without associated limb loss compared to the uninjured group, but injuries with associated limb loss were not [21]. Pain was also found to be associated with worse mental health outcomes in the cohort.

PTSD symptom clusters (e.g. hypervigilance, intrusive thoughts, avoidance behaviours and emotional numbing) were found to have specific, unique associations with risk factors of cardiovascular health, specifically cardiometabolic and haemodynamic effects/HRV [22, 23]. A similar investigation found that the five factors of the PTG-inventory (e.g. appreciation of life, new possibilities, personal strength, relating to others and spiritual change) were shown to be primarily associated with better cardiometabolic and haemodynamic indications of cardiovascular health [24]. However, both investigations identified secondary positive (for PTSD) and negative (for PTG) associations with cardiovascular health, indicative of a more complex interplay between mental health and cardiovascular functioning.

### Strengths and limitations

One of the strengths of the ADVANCE study cohort is the frequency-matched uninjured group. By following both groups, a greater understanding of the specific experience of serious physical combat injury can be inferred from our findings. Additionally, ADVANCE utilises multiple methods to assess a wide range of health outcomes. Data collected from medical assessments, clinical interviews and self-report questionnaire allows for both investigation of objective measures of health as well as subjective. Another strength of the study is the use of a participant panel. The panel provides a link to the opinions and views of the groups under investigation and are an invaluable resource in ensuring the participants have a voice in the future direction of the study.

Whilst the ADVANCE study is sufficiently powered to address the main hypotheses, a weakness of the study is that some groups are relatively small. At baseline, 162 personnel with an amputation injury participated. Though this represents a large percentage of all individuals who experienced injuries with associated limb loss in Afghanistan (62.6% of eligible participants) and is one of the largest cohorts of individuals with traumatic amputations worldwide, this group may provide challenges for statistical analysis of subgroups. Additionally, serious physical combat injuries range in complexity, from isolated broken bones/fragmentation injuries to complex injuries to different areas of the body, including multiple limb amputations. Consequently, there is considerable heterogeneity in the types of injuries sustained in the ADVANCE study cohort, which can cause some difficulty in assessing causal drivers of particular health outcomes.

An additional limitation of the cohort is that it relates only to the male experience of serious physical combat injury. Women were only allowed to take on front-line combat roles in 2014, the year of the Afghanistan withdrawal. As such, only a very small number of women experienced a serious physical combat injury during the conflict.

### Collaboration

Requests for access to the data for research purposes or suggestions for collaborations will be considered by the ADVANCE study project board and assessed on a case-by-case basis, subject to relevant UK Ministry of Defence and ethical approvals. For enquiries to access data or discuss potential collaborations, please visit our data discovery page https://www.advancestudydmrc.org.uk/data-discoverability/ or contact adv_data_team@imperial.ac.uk.

Acknowledgements.

## Supplementary Information

Below is the link to the electronic supplementary material.

Supplementary file1
