# Supplementary material for: Evaluating the physical and psychosocial impact of serious physical combat injuries in UK armed forces personnel-the ADVANCE cohort study
Source: Eur J Epidemiol. 2025 Sep 24;40(10):1263–71. doi: 10.1007/s10654-025-01300-2 (PMC12660447; doi:10.1007/s10654-025-01300-2)
Supplement: Supplementary file 1 — Supplementary file1 [file 10654_2025_1300_MOESM1_ESM.docx]

Supplementary materials 1: Baseline Demographics of Responders/Non-Responders to First Follow Up

|  | **Non-responder** | **Responder** |
| --- | --- | --- |
|  | Baseline characteristics (n=92) | Baseline characteristics (n=1053) |
| Age in years at baseline assessment (median (Interquartile range)) | **31 (28.5, 34)** | **34 (30, 37)** |
| Age in years at sampling (median (Interquartile range)) | **24 (21, 26.5)** | **25 (22, 29)** |
| Rank at sampling (n (%)) |  |  |
| Other rank/junior non-commissioned officers | **76 (82.6%)** | **678 (64.4%)** |
| Senior non-commissioned officers | **11 (12.0%)** | **242 (23.0%)** |
| Officer rank | **5 (5.4%)** | **133 (12.6%)** |
| Service (n (%)) |  |  |
| Naval Services (including Royal Marines) | **15 (16.3%)** | **146 (13.9%)** |
| Army | **75 (81.5%)** | **872 (82.8%)** |
| Royal Airforce | **2 (2.2%)** | **35 (3.3%)** |
| Type of injury (n (%)) |  |  |
| No injury (uninjured group) | **39 (42.4%)** | **527 (50.1%)** |
| Any injury (overall injured group) | **53 (57.6%)** | **526 (49.9%)** |
| Amputation-related injury | **21 (39.6%)** | **140 (26.6%)** |
| Non-amputation-related injury | **32 (60.4%)** | **386 (73.4%)** |
| New Injury Severity Score (median (Interquartile range)) | **17 (8, 33)** | **12 (5, 22)** |

Supplementary materials 2: Logistic Regression of Non-Response (e.g. did not attend/declined) for first follow up assessment

|  | **Odds Ratio (95% Confidence interval)** |
| --- | --- |
| **Injury group** |  |
| **Comparison** | Ref |
| ***Amputation injury subgroup*** | 1.76 (0.96, 3.04) |
| ***Non-amputation injury subgroup*** | 1.09 (0.62, 1.67) |
| **Rank at sampling** |  |
| **Junior Non-Commissioned Officer/Other Rank** | Ref |
| ***Senior Non-Commissioned Officer*** | 0.66 (0.31, 1.29) |
| ***Officer rank*** | 0.47 (0.16, 1.14) |
| **Age at baseline assessment** | 0.93 (0.88, 0.98) |
| *Bootstrapped using 1000 replications. Bias-corrected bootstraps reported. | |
